# Population dynamics in lesser black-backed gulls in the Netherlands support a North Sea regime shift

**DOI:** 10.1098/rsbl.2013.0127

**Published:** 2013-06-23

**Authors:** C. Luczak, G. Beaugrand, J. A. Lindley, J.-M. Dewarumez, P. J. Dubois, R. R. Kirby

**Affiliations:** 1 Université d'Artois, IUFM, centre de Gravelines, 40, rue Victor Hugo, BP129, Gravelines 59820, France; 2 Centre National de la Recherche Scientifique, LOG UMR 8187, Université Lille 1, Wimereux, France; 3 Sir Alister Hardy Foundation for Ocean Science, Plymouth, UK; 4 Marine Institute, Plymouth University, Plymouth PL4 8AA, UK

**Keywords:** climate change, food web, *Larus fuscus* graelsii, plankton, sea temperature, swimming crabs

Shamoun-Baranes & Camphuysen [1] made two main points in their critical appraisal of our recent article [2]: (i) that the *Larus fuscus* population increased in the Netherlands well before a mid 1980s regime shift in the North Sea and (ii) that population increases based on a simple prey type are difficult to imagine. These two comments give us the opportunity to deepen and complete our conclusions. Before we consider them, however, it is important to note that Shamoun-Baranes & Camphuysen [1] present conclusions outside the time window considered in our study, focus only on the Netherlands, and do not give any analysis of population trends.

With respect to the first point—that the *L. fuscus* population increased in the Netherlands well before a mid 1980s North Sea regime shift—it is important to highlight that the regime shift we referred to occurred in the mid-1990s. As our figure 2 [2] indicates clearly, the gull colonies in the Netherlands changed little during our study period from 1986 to 2000. Consequently, the first point made by Shamoun-Baranes & Camphuysen [1] has to be viewed as an idiosyncratic characteristic when considering spatial heterogeneity as a fundamental ecological property in ecosystem functioning [3], for which our analysis of gull populations in the broader North Sea region, allowed.

With respect to the second point—that population increases based on a simple prey type are difficult to imagine—Shamoun-Baranes & Camphuysen [1] support their argument with the observation that crustaceans have much lower energy value than fishes, which represented around 80 per cent of the gull's diet by mass. While we agree (even though we did not find these results in their cited reference [4]), the same study [4] reports that the swimming crab *Liocarcinus holsatus* is the third most important prey for *L. fuscus* in the Texel colony (20% in the 2005–2010 period), confirming *de facto* part of our findings. As shown by several authors [5,6], seabirds may be influenced by changes in both food quantity and quality, and they may affect different components of reproduction and survival: for example, fledging success (survival from hatching to fledging) shows a positive relationship with food quality, but not with food quantity [5]. In this regard, Shamoun-Baranes & Camphuysen [1] omitted to mention a key study by Schwemmer & Garthe [7], which showed that swimming crabs—*Liocarcinus* sp*.*—were a major dietary item during both the egg-stage and the chick-rearing period, when the number of breeding pairs of *L. fuscus* were increasing exponentially along the German coast in the 1990s (figure 2 in [2]). During the breeding season, birds require specific nutrients in their diet [8] and Schwemmer & Garthe [7] suggested that swimming crabs—*Liocarcinus* sp.—may be a valuable source of calcium for both eggshells and the bone development of chicks. Interestingly, the observations by Schwemmer and Garthe may have also provided an answer to Camphuysen [9], who concluded that the foraging range of *L. fuscus* further out to sea could not be explained fully by either a change in the abundance of fishing vessels or the avoidance of herring gulls (*Larus argentatus*), and that it must, therefore, involve another unknown offshore food resource. Shamoun-Baranes & Camphuysen [1] only referred to their own local study [9] on the importance of fishing discards in the gull's diets, whereas swimming crabs found in the *L. fuscus*'s diet are obtained by natural feeding [4,7]. One hypothesis proposed by Schwemmer & Garthe [7] to support their findings was an increase in the abundance of *Liocarcinus* sp., which we confirmed in our study [2].

To conclude, the observations of Shamoun-Baranes & Camphuysen [1] on a single population of *L. fuscus* from the Netherlands most likely indicate that they were at the wrong place (idiosyncratic results owing to spatial scaling and heterogeneity), at the wrong time period (*ante* 1989), while also failing to consider food quality as an important parameter in food selection. Although Lindley *et al*. [10] showed that the increase in decapods (dominated by swimming crabs of the sub-family Polybiinae) was a key component of the trophic amplification of hydroclimatic change and the development of a new North Sea ecosystem dynamic regime [11,12], we do agree with Shamoun-Baranes & Camphuysen [1] that a deeper exploration of the drivers influencing gull population dynamics at several spatial and temporal scales is needed, as it is for any complex adaptive system.
